# Ionic direct current modulation evokes spike-rate adaptation in the vestibular periphery

**DOI:** 10.1038/s41598-019-55045-6

**Published:** 2019-12-12

**Authors:** Marco Manca, Elisabeth Glowatzki, Dale C. Roberts, Gene Y. Fridman, Felix P. Aplin

**Affiliations:** 1 Department of Otolaryngology Head and Neck Surgery, Johns Hopkins University, Baltimore, Maryland 21205 United States; 2 Department of Neuroscience, Johns Hopkins University, Baltimore, Maryland 21205 United States; 3 Department of Electrical and Computer Engineering, Johns Hopkins University, Baltimore, Maryland 21205 United States; 4 Department of Biomedical Engineering, Johns Hopkins University, Baltimore, Maryland 21205 United States

**Keywords:** Translational research, Biotechnology, Inner ear

## Abstract

Recent studies have shown that ionic direct current (iDC) can modulate the vestibular system *in-vivo*, with potential benefits over conventional pulsed stimulation. In this study, the effects of iDC stimulation on vestibular nerve fiber firing rate was investigated using loose-patch nerve fiber recordings in the acutely excised mouse crista ampullaris of the semicircular canals. Cathodic and anodic iDC steps instantaneously reduced and increased afferent spike rate, with the polarity of this effect dependent on the position of the stimulating electrode. A sustained constant anodic or cathodic current resulted in an adaptation to the stimulus and a return to spontaneous spike rate. Post-adaptation spike rate responses to iDC steps were similar to pre-adaptation controls. At high intensities spike rate response sensitivities were modified by the presence of an adaptation step. Benefits previously observed in behavioral responses to iDC steps delivered after sustained current may be due to post-adaptation changes in afferent sensitivity. These results contribute to an understanding of peripheral spike rate relationships for iDC vestibular stimulation and validate an *ex-vivo* model for future investigation of cellular mechanisms. In conjunction with previous *in-vivo* studies, these data help to characterize iDC stimulation as a potential therapy to restore vestibular function after bilateral vestibulopathy.

## Introduction

The use of slowly modulated direct current (DC) to stimulate the vestibular system has a long history as a scientific, diagnostic and therapeutic tool to probe and understand vestibular function in normal function and pathophysiology^1,2^. Typically, DC, as ‘galvanic stimulation’, is applied non-invasively across the mastoid processes of the head. In animal experiments, electrodes have also been inserted into or close to the inner ear to increase the specificity of the resultant electric field. Galvanic stimulation results in a generalized activation or inhibition of all vestibular end organs^3–8^. Therefore, an invasive stimulation approach is necessary in order to activate specific vestibular canals. An evolution of this concept is the focused, safe delivery of ionic direct current (iDC) separately into each vestibular semicircular canal through an electrolyte-filled microcatheter with the intent to restore functional vestibular sensation for patients suffering from bilateral vestibular dysfunction^9–11^. iDC has an advantage over traditional pulse-based neuro-stimulation, in that iDC can excite or inhibit the vestibular system depending on the polarity of the stimulating current^12^. This is important because information about the direction and magnitude of head rotation is encoded in the vestibular afferents as either an increase or decrease around a spontaneous firing rate^13^.

Based on vestibular-ocular reflex responses to iDC stimulation in the awake chinchilla *in-vivo*, it was recently shown that an iDC-based neuro-modulation approach is functionally feasible and may have benefits over a comparable pulsatile methodology^9,10^. However, these studies suggest that the relationship between iDC and vestibulo-ocular reflex (VOR) output is dependent on whether short (150 ms) current steps were delivered independently or after long (=>1 minute) “adaptation” steps^9^. These *in-vivo* studies raise questions as to how iDC affects the peripheral neuroepithelium. The aim of this study was to determine the effects of iDC stimulation on the vestibular afferent fibers.

Although some previous studies associated with galvanic or direct current neuromodulation hint at expected the outcomes, given the difference in iDC delivery mechanism, location, and assays, it is not immediately clear how well their conclusions apply the use of iDC in the vestibular system. It has been well established from the field of trans-cranial DC stimulation (tDCs) that the relative distribution and magnitude of an artificially generated electric field dramatically influences the relationship between stimulus and neural response^14–17^. The interaction between electrode placement, neural morphology and non-neural tissue impedances can create complex current paths that result in a non-homogeneous effect on the target tissue. Electrode location has also been identified as a primary source of heterogeneity between galvanic stimulation studies^3,5,7^. Galvanic stimulation is typically delivered non-invasively across the mastoid processes. While previous work on galvanic stimulation noted the possibility for, it did not characterize adaptation in response to constant current DC stimulation^3,6^. iDC stimulation differs from galvanic stimulation experiments in that the stimulating electrode is in a very different position, located directly adjacent to or within the vestibular sensory epithelia. Although some galvanic stimulation studies have examined the effect of current delivered internally at the round window or inner meatus^3,5,6^, this is still comparatively distant. An experimental validation is necessary to confirm whether iDC stimulation produces comparable neural responses to the more diffuse galvanic stimulation. A proper exploration and characterization of iDC-evoked afferent activation would be enhanced by an *ex-vivo* methodology that removes these confounds associated with non-uniform current delivery through the skull and soft tissues of the head.

To directly investigate the effects of iDC stimulation on vestibular nerve fiber activity in the crista ampullaris of the semicircular canals, an *ex-vivo* approach was developed. Compared to previous *in-vivo* experiments, this method will allow for the future study of cellular mechanisms involved in vestibular stimulation. As such, it was first necessary to validate this model against previous data from studies using *in-vivo* iDC or galvanic stimulation. iDC stimulation was performed in the acutely excised mouse crista ampullaris, while monitoring afferent firing rates with extracellular loose-patch recordings from vestibular nerve fiber endings. In this experimental setting, the effects of stimulator position and short and long duration iDC step modulation on afferent activity were examined. To provide a point of comparison to the previous galvanic stimulation literature, the effects of sinusoidally modulated current was also investigated. The response following sustained iDC delivery showed an adaptation effect that influenced sensitivity to further iDC-evoked afferent responses. The peripheral spike rate relationships for iDC vestibular stimulation in the preparation used here were similar to those found with galvanic stimulation. These data provide important insights for future design of iDC stimulation paradigms and validate the *ex-vivo* preparation as a model system that will allow further investigation of underlying intracellular mechanisms of DC vestibular stimulation.

## Results

### Monitoring vestibular nerve fiber firing rate in response to iDC current stimulation in the crista *ex vivo*

**Figure 1:**
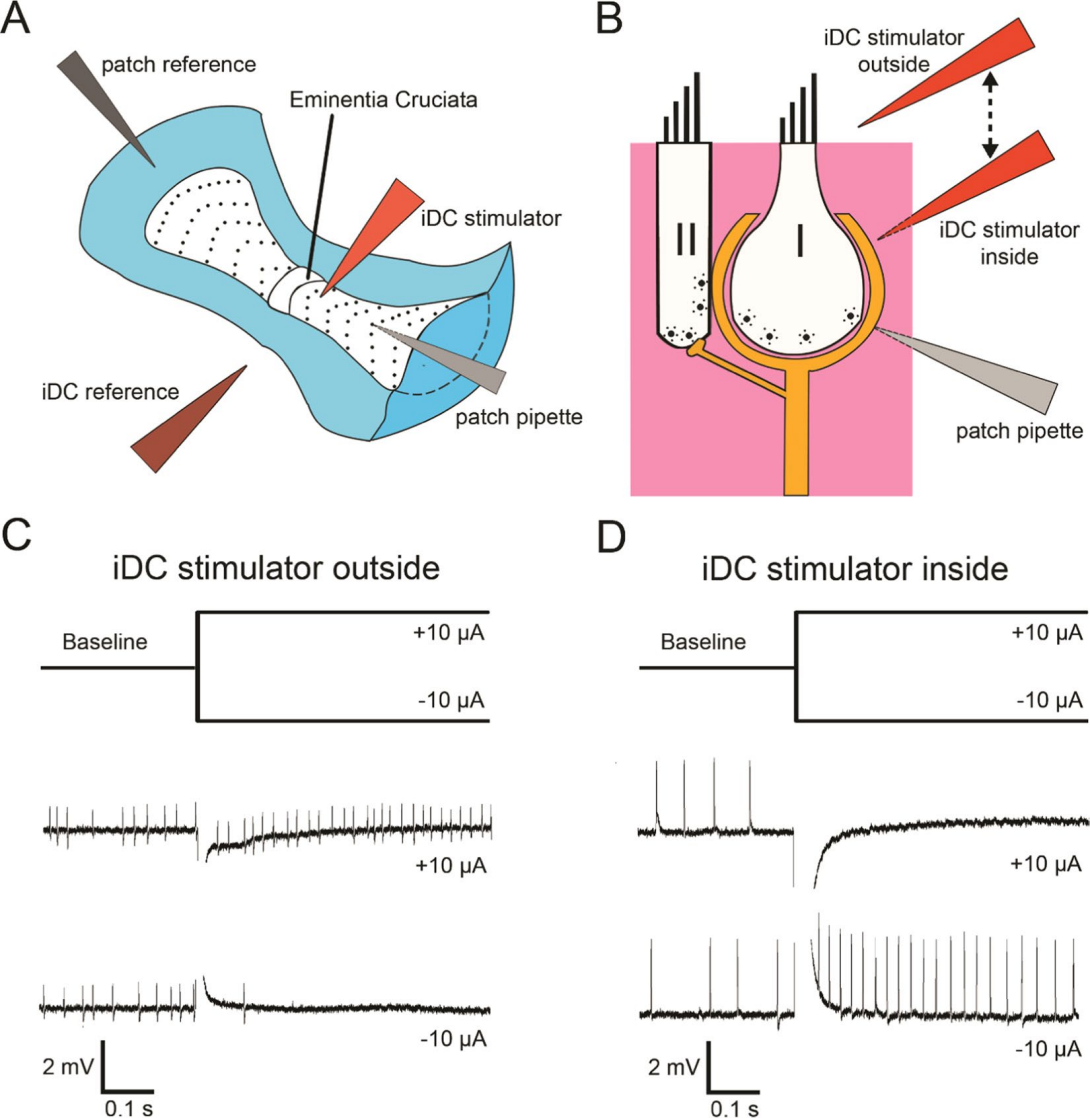
Afferent responses in the vestibular crista *ex vivo* are dependent on iDC stimulator position. (**A**) Schematic drawing of an anterior crista ampullaris with approximate location of iDC stimulator electrode and patch pipette. Modified from Tavazzani *et al*.^39^. (**B**) Schematic drawing of the afferent innervation of type I and type II hair cells in the vestibular crista showing the position of the recording and stimulation electrodes in two configurations (inside and outside of the tissue). (**C**,**D**) Stimulus traces and representative afferent loose-patch recordings. When the iDC stimulator is outside the epithelium (**C**), there is an excitatory response to anodic (+) iDC steps and inhibitory response to cathodic (−) iDC steps (n = 9). When the iDC stimulator is inside the epithelium (**D**), the effect is reversed (n = 28).

To provide a characterization of the timing of action potential firing and of changes in firing rate during iDC stimulation of the vestibular periphery, AP firing rate was monitored with loose-patch recordings from vestibular nerve fiber endings at 37 °C in the anterior canal of the acutely excised mouse crista (P18–30) (Fig. 1A). In the vestibular periphery, two types of hair cells (type I and type II) (Fig. 1B) serve as sensory cells that convert the mechanical movements of the hair bundles in response to head motion into changes in hair cell receptor potential. This then leads to subsequent changes in firing rates in vestibular nerve fibers via hair cell synaptic transmission. These afferent fibers receive input via type I hair cell-ensheathing calyx endings and via type II hair cell-contacting bouton endings. Type II hair cells also provide input directly to calyces via direct wall-to-wall synapses. Most afferent fibers receive inputs via multiple bouton and calyx endings, and some may just receive bouton-only or calyx-only inputs. The timing of action potential firing recorded with a patch pipette on the calyx structure of an afferent fiber is thought to be the result of the combined inputs the recorded fiber receives from the connected presynaptic hair cells (Fig. 1A,B).

### Afferent responses are dependent on iDC stimulator position

Previous studies have shown that the location and amplitude of DC stimulation is important for determining the afferent response^3,4,7^. To examine the effect of location, the iDC stimulator was positioned in one of two different configurations: either outside and above the vestibular sensory epithelium, or inside the tissue at hair cell level (Fig. 1B). In both cases, the stimulation pipette was positioned between 75–200 µm from the patch pipette. When the stimulator was outside of the tissue, anodic (+) current steps increased the afferent spike rate, while cathodic (−) steps reduced the spike rate (Fig. 1C). Conversely, when the stimulator was inside the tissue at the level of the calyx, anodic steps reduced spike rate and cathodic steps increased spike rate (Fig. 1D). This effect was consistently reproduced across experiments (n = 9 outside; n = 28 inside). In one afferent recording, the response could be inverted by moving the iDC stimulator from outside to inside the tissue during recording. All further experiments were performed with the iDC stimulator in the tissue (second configuration) as the relationship between current polarity and spike rate response matched previous behavioral experiments in chinchilla^9,10^.

### Afferent responses are dependent on iDC stimulation amplitude

**Figure 2:**
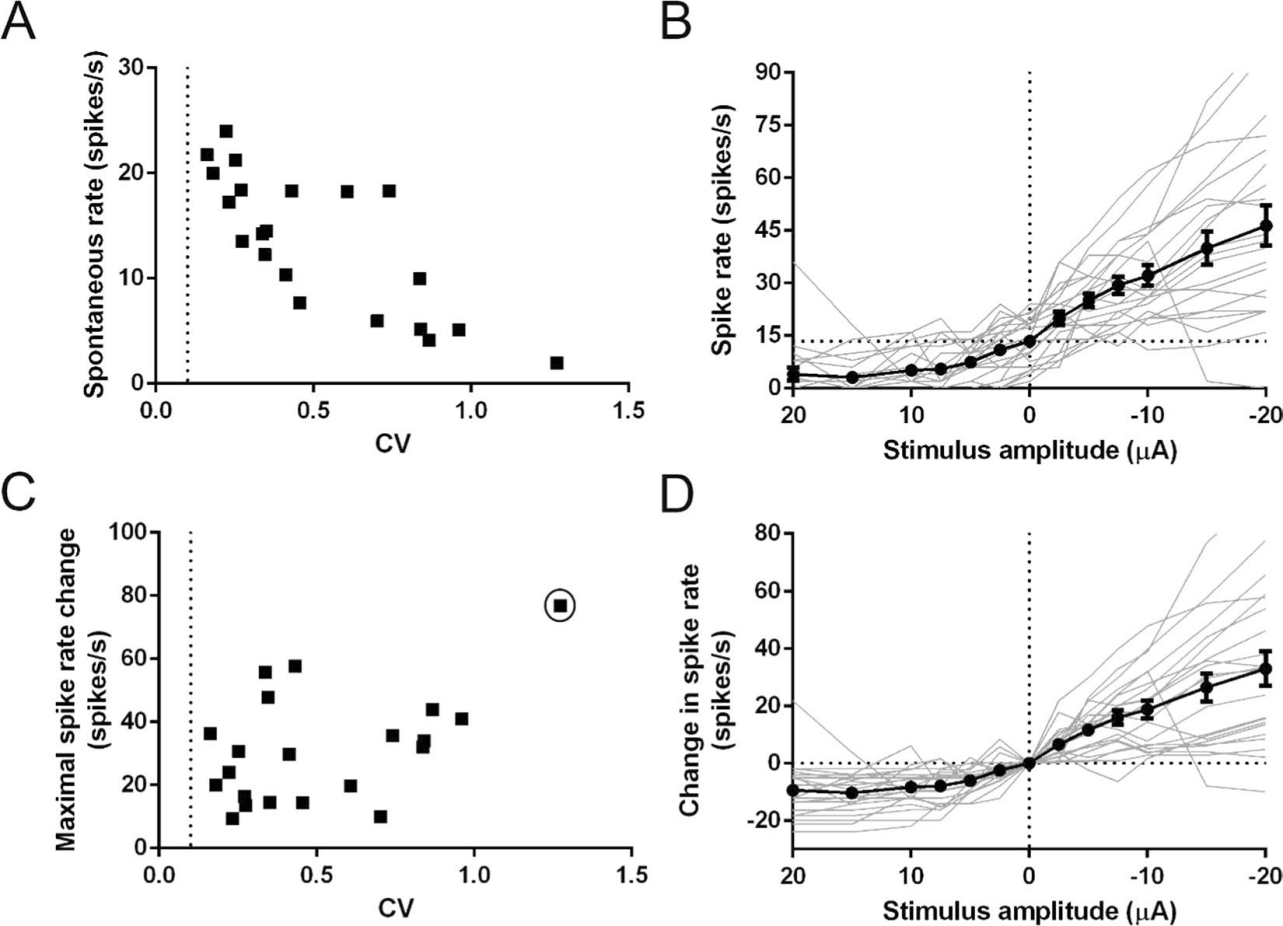
Afferent responses are dependent on iDC stimulation amplitude. (**A**) CV and spontaneous rate distribution of the analyzed population. There was an inverse correlation between CV and spontaneous rate (Pearson’s; r = −0.7850; p < 0.0001; n = 21 recordings). (**B**) Individual (grey) and population (black) spike rates in response to anodic (+) and cathodic (−) 2 s-long iDC steps. iDC stimulation resulted in a change of the mean spike rate (Friedman; p < 0.0001; n = 21 recordings). Compared to the spontaneous spike rate (at 0 µA stimulus intensity), the anodic current steps reduced the spike rate, whereas cathodic current increased the spike rate. (**C**) CV and maximal spike rate change distribution of the analyzed recordings. Increased CV correlated to an increased maximal spike rate change (Pearson’s; r = 0.4879; p = 0.0248; n = 21 recordings) but this was not significant if the high CV outlier (circled) was excluded (p = 0.3182; n = 20 recordings). (**D**) Individual (grey) and population (black) spike rate change in response to anodic (+) and cathodic (−) iDC steps. Change in spike rate also varied significantly with change in amplitude (Friedman; p < 0.0001; n = 21 recordings). Cathodic iDC steps elicited a greater absolute spike rate change on average than anodic iDC steps (Wilcoxon; p < 0.0001; n = 21 recordings). Error bars are ± SEM. Dotted line at CV = 0.1 in (**A**,**C**) shows the cutoff between regular and irregular cells^21^.

Before applying iDC stimulation, spontaneous firing was monitored in individual recordings. Spontaneous rates ranged from 1.96 spikes/s to 24 spikes/s with a mean rate of 13.44 spikes/s. This is lower than previously reported spontaneous rates *in-vivo*^18^ but comparable to previous *ex-vivo* preparations^19^. Lower spike rates may be due to the higher Ca^2+^ concentration at the hair cell stereocilia than the physiological norm in the *ex-vivo* preparation, leading to a reduced number of open mechano-transduction channels and resulting in hyperpolarized hair cell membrane potentials^20^. Spike timing variability (normally discriminated using the coefficient of variation, CV) is an important determinant of cell type in the vestibular periphery. Vestibular afferents are divided into two loose classes (irregular and regular) based on their spike timing variability^21^. Figure 2A shows the pre-stimulation spontaneous rate in each recording as a function of the spike timing variability (coefficient of variation; CV). Higher CV correlated with lower spontaneous rates (Pearson’s; r = −0.7850; p < 0.0001; n = 21 recordings). The range of CVs (0.16 to 1.27) was similar to previously reported CV ranges in the mouse *in-vivo* (~0.02 to 1.5)^18^. Based on spike rate and CV, no distinctive clustered responses were found that would allow to cleanly discriminate irregular versus regular afferent fibers; likely due to the lack of low (CV < 0.1) afferent recordings. Therefore, the continued analysis was performed on the dataset as a whole.

To measure the afferent response to iDC modulation, 2-second-long cathodic or anodic iDC steps with interstep periods of 10 seconds were applied. Current was stepped sequentially across an increasing range of ±2.5 to 20 µA. Spontaneous rates were quantified in a 60 second window before stimulation, and onset spike rates during stimulation in a 50–550 ms window after the stimulation pulse onset, to avoid the stimulus artefact (see Fig. 2). Increasing anodic/cathodic stimulus intensity resulted in larger inhibitory/excitatory effects on mean afferent onset spike rate, respectively (Fig. 2B, black trace; Friedman; p < 0.0001; n = 21 recordings). Afferent fibers with a low spontaneous rate were often silenced as a result of increased anodic current (Fig. 2B; see grey traces representing individual recordings). This effect might be due to or exaggerated by the lower spontaneous rates in the *ex-vivo* preparation compared to *in-vivo*. To normalize the variation between spontaneous rate baselines, responses were analyzed as a function of spike rate change (spike rate response versus pre-stimulation spontaneous rate). Figure 2C shows the relationship between the maximal spike rate change elicited during iDC stimulation versus the pre-stimulus CV for each recording. Higher CV correlated with larger maximal rate changes (Pearson’s; r = 0.4879; p = 0.0248; n = 21 recordings) but this trend was reliant on a high CV outlier and was not significant if this was excluded (p = 0.3182; n = 20 recordings). Across the range of iDC amplitude steps (Fig. 2D) change in spike rate also varied significantly with change in amplitude (Friedman; p < 0.0001; n = 21 recordings). Cathodic iDC steps elicited a greater absolute spike rate change on average than anodic iDC steps (~ 2–3 fold) (Wilcoxon; p < 0.0001; n = 21 recordings). This held even when units that were silenced by anodic iDC were removed from the statistical analysis (Wilcoxon; p < 0.0195; n = 10). Overall, iDC modulates vestibular afferent responses with an effect dependent on electrode location, stimulus polarity and stimulus amplitude.

### Responses to sinusoidal current modulation show high-pass characteristics

**Figure 3:**
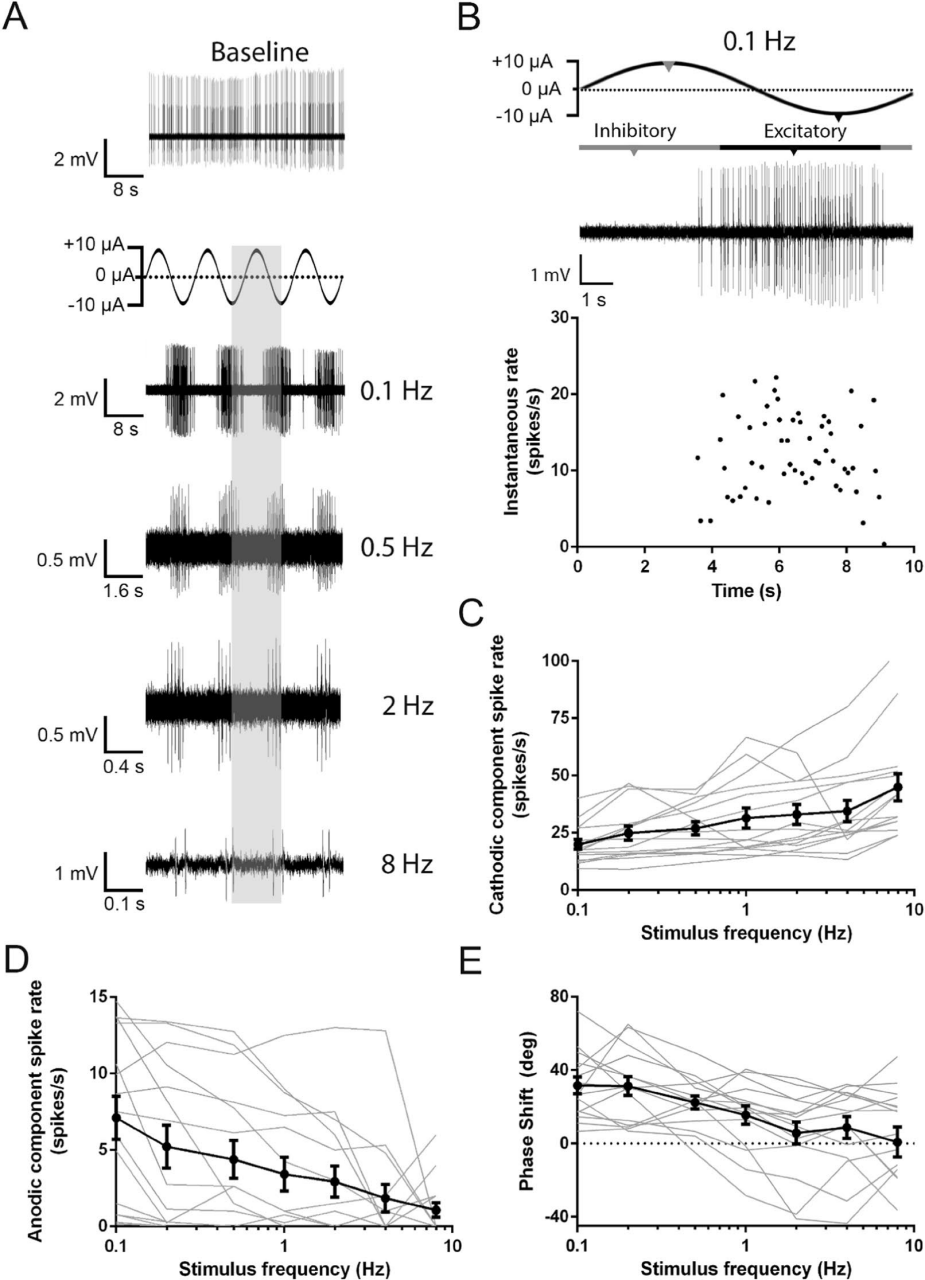
Characterization of firing rate and phase responses to sinusoidal iDC stimuli. (**A**) Stimulation waveform and representative afferent responses to a ±10 µA sinusoidal current across a range of frequencies. The shaded vertical column indicates the single cycle of the stimulus. The responses are aligned to the single stimulus cycle to observe the phase relationship of the responses to the different stimulation frequencies. (**B**) Enlarged 0.1 Hz responses from (**A**) with accompanying diary plot of instantaneous rate, to show the median spike calculation to determine excitatory (black notches)/inhibitory (grey notches) response phase relative to sinusoidal stimulus phase. (**C**–**E**) Individual (grey) and average (black) excitatory phase **(C**) and inhibitory phase (**D**) spike rate responses across stimulus frequencies. As frequency increased, the excitatory phase spike rate increased (Friedman; p < 0.0001; n = 15 recordings) and the inhibitory phase spike rate decreased (Friedman; p < 0.0001; n = 15 recordings). (**E**) Individual (grey) and average (black) phase shift of the excitatory spike rate response in relation to the cathodic stimulus waveform across different frequencies. There was a phase lag at low frequencies that decreased as stimulation frequency increased (Friedman; p > 0.0007; n = 15 recordings). Error bars are ± SEM. Representative traces were filtered with a Gaussian bandpass filter (5 Hz −2.5 kHz) to better display the data.

In previous studies, the effects of galvanic stimulation in the vestibular system have typically been probed using sinusoidal stimulation^4^. For comparison, afferent response characteristics to iDC were examined using ±10 µA sinusoidal stimulation waveforms applied across a physiologically relevant frequency range (0.1 to 8 Hz)^22^. Sinusoidal current modulation resulted in distinctive components of afferent firing, with an excitatory and inhibitory component compared to spontaneous firing (Fig. 3A,B). During excitation, the rate changes did not follow a sinusoidal waveform, suggesting a non-linear response (Fig. 3B diary plot). To allow for an analysis of frequency dependent timing of the response, a 180 degree excitatory component was defined by the occurrence time of the middle spike in the burst (Fig. 3B, arrow), and the inhibitory component was assigned the remaining 180 degrees. Spike rate was analyzed separately during each component (Fig. 3C–E). Compared to the spontaneous spike rate baseline, afferents appeared to have higher sensitivities at higher sinusoidal frequencies. There was an increased spike rate during the excitatory (cathodic) component (Fig. 3C; Friedman; p < 0.0001; n = 15 recordings) and reduced spike rate during the inhibitory (anodic) component (Fig. 3D; Friedman; p < 0.0001; n = 15 recordings). The occurrence time of the middle spike was not always aligned to the peak of the sinusoidal waveform (Fig. 3E). Instead, at low frequencies there was a tendency for the response to shift toward the highest rate of change in the sinusoidal stimulus away from the peak of the sinusoid, i.e. there was a “phase lead”, that was reduced as stimulation frequency increased (Friedman; p = 0.0007; n = 15 recordings). Signal attenuation and a phase lead at low frequencies suggests that afferent responses to iDC display high-pass characteristics.

### iDC stimulation induces spike rate adaptation

**Figure 4:**
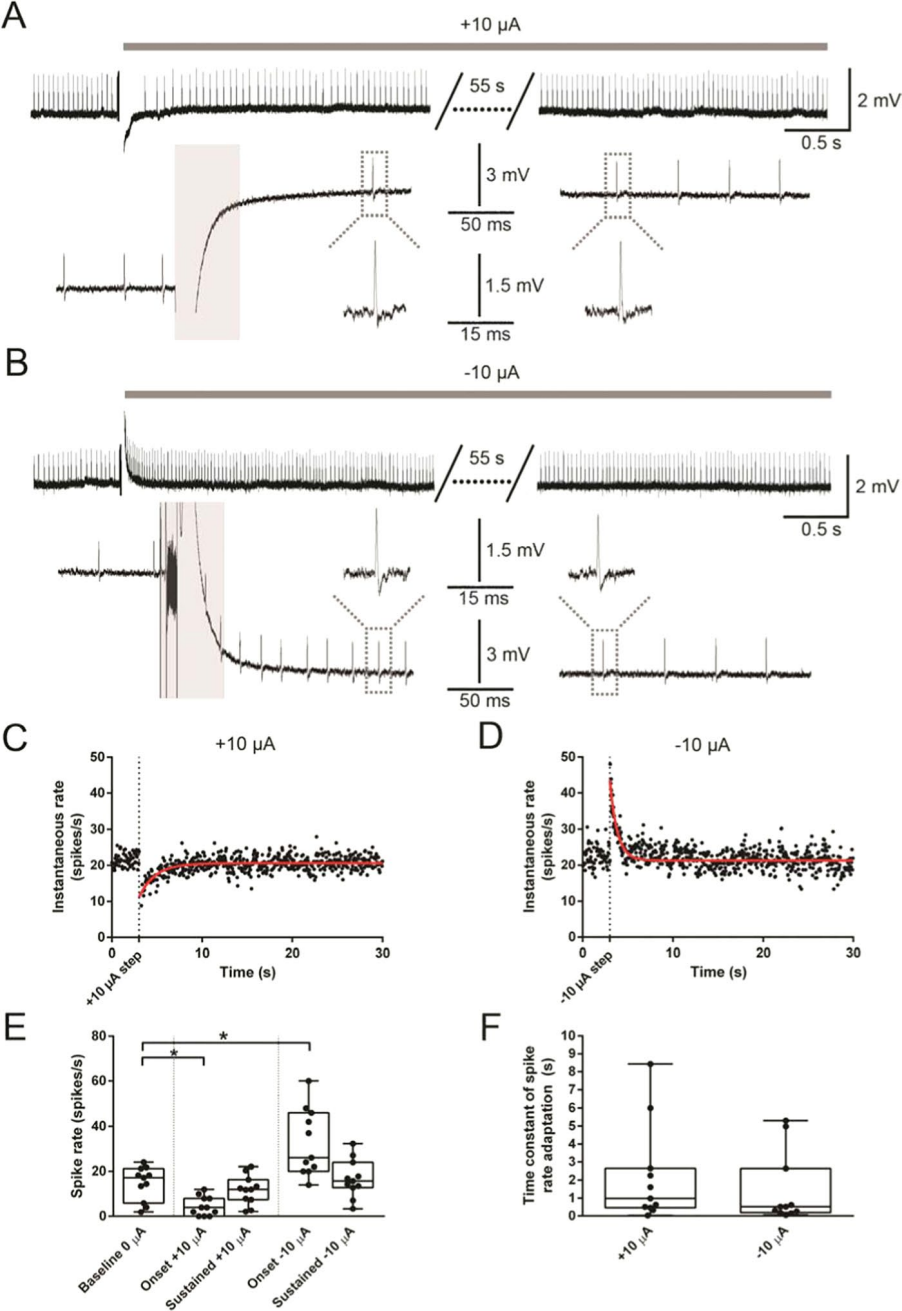
iDC stimulation induces spike rate adaptation. (**A**,**B**) Representative traces showing an afferent response to a +10 µA and −10 µA 60 s long iDC step, at 3 magnifications. The grey bar indicates the 50 ms period during the artifact where analysis was blanked. Dotted lines indicate representative spikes that were further expanded. (**C**,**D**) Diary plots of instantaneous spike rate versus time for the recordings shown in (**A**,**B**). Each data point reflects 1/inter-event interval. Red lines show one-phase exponential fits used to determine time constants of spike rate adaptation. (**E**) box and whiskers plot showing the spontaneous rate (60 seconds pre-stimulus), onset spike rate (first 50–550 ms after stimulus onset), and sustained spike rate (10–60 s after stimulus onset) during a 60 s long ±10 µA step. Compared to the baseline spike rate, at onset, anodic and cathodic steps reduced or increased the spike rate, respectively (Friedman; p < 0.0001; Dunn’s multiple comparisons; −10 µA onset: p = 0.28; +10 µA onset: p = 0.0061; n = 11 recordings) but sustained spike rates were not significantly different from baseline (Dunn’s multiple comparisons; −10 µA sustained: p > 0.9999; +10 µA sustained: p > 0.9999; n = 11 recordings). (**F**) Box and whiskers plots showing the distribution of spike rate adaptation time constants to 1 min ± 10 µA iDC steps. There was no significant difference in the time course of spike rate adaptation between cathodic and anodic steps (Wilcoxon; p = 0.5195; n = 11 recordings). In (**A**,**B**) for better display of the long duration data, the traces were filtered with a Bessel (8 poles) 50-Hz high pass filter.

Spike rate adaptation in response to constant stimulation currents has been commonly reported in the nervous system, including in the central vestibular system^23^. Existing literature on galvanic stimulation has identified a possible adaptation effect but did not attempt to quantify the afferent response to long-duration DC steps^3,10^. To investigate the potential for time-dependent changes in the afferent response to iDC stimulation, 1 min long iDC steps of ±10 µA were applied, and afferent response rates were monitored (Fig. 4A,B). Spike rates showed an initial decrease or increase for anodic or cathodic steps respectively, but this effect adapted over time (Fig. 4A–D). The pre-stimulus spontaneous rate (60 seconds before stimulus onset) was compared to the ‘onset’ spike rate (first 50–550 ms after stimulus onset) and the ‘sustained’ spike rate (10–60 s after stimulus onset). Recordings are shown at extended time scales in Fig. 4A,B. Illustrating that after the excluded window during the stimulation artifact (grey bars), action potentials could be clearly identified by their waveform within the analyzed time window. Compared to the mean pre-stimulus spike rate of 14.63/s, an anodic or cathodic iDC step resulted in a mean −10.09/s decrease or 1 + 18.01/s increase in the ‘onset’ spike rate, respectively (Friedman; p < 0.0001; Dunn’s multiple comparisons; −10 µA onset: p = 0.28; +10 µA onset: p = 0.0061; n = 11 recordings) but this effect was not maintained for ‘sustained’ spike rates, where the median spike rates (16.84/s for anodic, 11.72/s for cathodic) were not significantly different from the pre-stimulus spike rate (Dunn’s multiple comparisons; −10 µA sustained: p > 0.9999; + 10 µA sustained: p > 0.9999; n = 11 recordings).

The time constant of spike rate adaptation was determined by a single-phase exponential fit (Fig. 4C,D, red lines). Single phase exponentials were a better fit than two-phase exponentials (sum-of-squares F test; p < 0.001; n = 11 recordings) suggesting a single dominant time constant. The time constant associated with the response decay varied considerably between individual trials (Fig. 4F) but was not significantly different between anodic and cathodic iDC with medians of 0.99 s and 0.53 s respectively (Wilcoxon; p = 0.5195; n = 11 recordings). Spontaneous rates before and after the conclusion of the iDC protocol were not significantly different (paired t-test; d.f. = 8; p = 0.4156; n = 11 recordings).

### Changes in iDC-induced spike rate activation after spike rate adaptation

**Figure 5:**
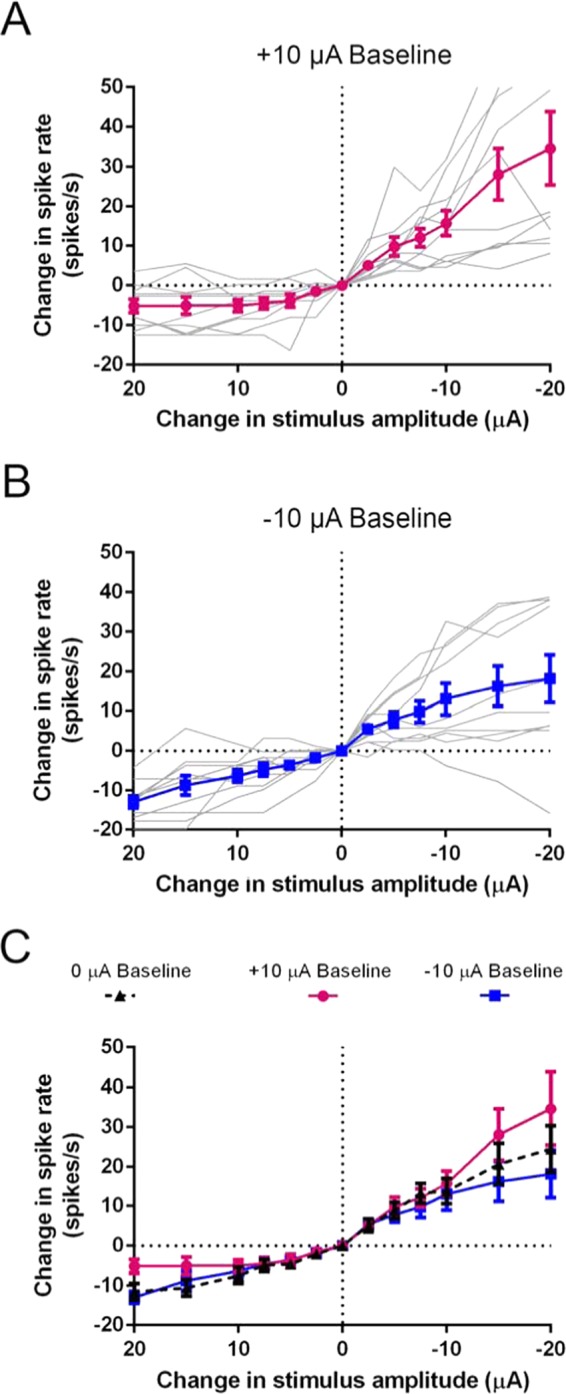
Changes in iDC-induced spike rate activation after spike rate adaptation. (**A**,**B**) Individual (grey) and population (coloured) spike rate change in response to anodic (+) and cathodic (−) 2-s long iDC steps (protocol as in Fig. 2) after adaptation to a constant +10 µA **(A)** or −10 µA (**B**) baseline, applied for 60 s before steps. (**C**) Mean spike rate change in response to anodic (+) and cathodic (−) iDC across different baselines (+10 µA, 0 µA and −10 µA). There was a significant interaction between baseline and step amplitude (2way RM ANOVA; p = 0.0382; n = 10 recordings). At the greatest cathodic intensity (−20 µA), when compared to the 0 µA baseline control, the elicited change in spike rate was larger for the +10 µA baseline (Holm-Sidak multiple comparisons; p = 0.0016) and smaller for the −10 µA baseline (p = 0.0288). At the greatest anodic intensity (+20 µA), the change in spike rate was smaller for the +10 µA baseline (p = 0.0222) but not significantly different for the −10 µA baseline (p = 0.3129). Error bars are ±SEM.

Previous work in the chinchilla showed that tonic iDC baselines influenced the behavioral VOR response to subsequent iDC steps^9^. Therefore, the response to iDC steps during the application of constant iDC holding baselines was investigated (Fig. 5). After holding the preparation at ±10 µA for 60 s, iDC steps were delivered as per Fig. 2 (Δ 0.25 to 20 µA) but now offset around the ongoing ±10 µA iDC baseline. Spike rate responses to iDC steps during anodic (Fig. 5A) and cathodic (Fig. 5B) baselines were qualitatively similar. However, when means were compared between baselines (Fig. 5C) there was a significant interaction between baseline and step amplitude (2way RM ANOVA; d.f. = 20; p < 0.0013; n = 10 recordings). When compared to the 0 µA baseline condition, during the −20 µA step there was a significant increase in spike rate for the anodic (+) baseline and significant decrease for the cathodic (−) baseline (Holm-Sidak multiple comparisons; d.f. = 216; +10 µA: p = 0.0002; −10 µA: p = 0.0155; n = 10 recordings). During the +20 µA step there was a significant decrease in spike rate for the anodic baseline but no significant difference compared to controls for the cathodic baseline (+10 µA: p = 0.0307; −10 µA: p = 0.5803; n = 10 recordings). These data suggest that iDC holding baselines can affect response properties of the afferent and induce higher magnitude changes in iDC stimulation.

## Discussion

The purpose of this study was to validate and characterize the peripheral vestibular response to iDC in an *ex-vivo* preparation of the mouse crista. Changes in spike rate are found to be proportional to modulation polarity and amplitude for brief (500 ms) stimulation durations. These results closely match previous findings by Goldberg and others^3–5^, suggesting that data obtained using an *ex-vivo* vestibular preparation such as described here are compatible with the broader galvanic stimulation literature. Moreover, sustained (60 s) iDC stimulation results in spike rate adaptation back to the initial spontaneous baseline of the afferent. Further stimulation post-adaptation appears to modify the relationship between spike rate and iDC steps at higher stimulation amplitudes. Together, these data show that the vestibular afferent response to iDC modulation is dependent on multiple factors beyond stimulation amplitude. These results provide insights into the heterogeneity of responses to galvanic stimulation in the broader literature and, most importantly, are vital for the continued development of iDC stimulation paradigms.

Experiments that examine the effect of transcranial DC stimulation (tDCS) have extensively characterized the response of cortical neural networks to DC fields^15–17,24^. These studies have repeatedly demonstrated that the polarization of individual neurons and even morphological regions within the same neuron is dependent on the individual position and morphology of the neural cell in relation to the density and orientation of the electric field^14,15,17,25^. As such it is not surprising that iDC in the vestibular periphery shows a similar effect. However, tDCS has several key differences to iDC stimulation in the vestibular periphery that require careful interpretation. The neural effects of tDCS are likely driven by subthreshold circuit level modulation due to low, diffuse electric field densities at the neural substrate^15,17^. This is substantially different to iDC stimulation, where the DC fields are being generated invasively at close proximity (100 s of microns) at much higher densities, and without spread through the bone and soft tissues of the head. Furthermore, the morphology of the vestibular periphery is substantially different to cortical morphology, with a high-impedance sensory epithelia, unique sensory synapse and large distance between the axon and monopolar soma. In contrast to tDCs, galvanic stimulation is thought to directly drive neural responses in the vestibular system and is thus a closer point of similarity to iDC stimulation^7^.

Galvanic DC studies in the vestibular system have variously demonstrated vestibular excitation using both cathodic and anodic stimulation^3,4,6–8^. Results shown here suggest that the reported differences in cathodic versus anodic effects could be due to different electrode positioning. Changing the electrode position in the preparation likely affects the orientation of the vestibular afferent relative to the electrical field. In fact, afferent responses were influenced by the iDC stimulator position, with cathodic current generating a net excitatory effect on the afferent when within the epithelium and a net inhibitory effect when external to the tissue (and vice versa for anodic current). Interestingly, results from the *ex-vivo* preparation with the stimulation pipette inside the tissue resemble previous data in chinchilla *in-vivo*, where an electrode within the semicircular canal elicited excitatory VOR during cathodic stimulation^9^. The surgical approach for iDC delivery in the chinchilla results in a collapsed membranous labyrinth around the ampulla and therefore subsequent stimulation within the canal may flow around the canal and through the tissue. Similarly, Goldberg *et al*.^3^ hypothesized that a cathodal electrode above the tissue might result in an inward, inhibitory current at the afferent while an electrode positioned in the perilymph (equivalent to the tissue) might result in an outward, excitatory current. In summary, findings from the *ex-vivo* study here support the assertion of Kwan *et al*.^7^ that electrode location plays a major role in the effect of galvanic or invasive DC on the vestibular epithelia. As such, experimental differences in positioning may explain the heterogeneity in galvanic responses reported across the literature.

Vestibular afferents are typically classified by the regularity of their spontaneous rate^26–28^. Coefficient of variation (CV) is used to discriminate between these groups, with a transformed measure (CV*) that normalizes this measure for neurons characterized by different mean spontaneous activity^3^. Due to a small population size and lower spontaneous rate, in this study afferents could not be normalized to CV* in a meaningful manner. Lower spontaneous rates are likely a result of the *ex-vivo* preparation^19^ as *in-vivo* mouse preparations have higher resting rates^18^. However, in accordance with previous literature, afferents with a lower CV tended to have lower spontaneous rates^26^.

The spike rate responses recorded in this study largely match the VOR eye movement responses to analogous iDC stimuli described in a previous *in-vivo* study in the chinchilla^9^. Both datasets showed an increased sensitivity to stepped cathodic iDC over anodic iDC with a similar ratio (~3:1). This trend is in agreement with previous literature for irregular (CV > 0.1) neurons^8,26^ and may result from mechanisms underlying physiological inhibitory/excitatory asymmetry of high CV neurons exhibited for the more natural rotational stimuli^8,29,30^. Responses to sinusoidal currents displayed high-pass characteristics and were strikingly similar to galvanic responses reported in vestibular afferents of *Xenopus laevis* tadpoles suggesting that these methodologies are comparable^4^. Such high-pass characteristics make intuitive sense in light of the finding that long duration stepped currents (60 s) created spike rate adaptation, and the time constants of response decay (~0.1 to 10 s) are within the same range as the delivered sinusoids (0.1 to 8 Hz). In the literature more broadly, a higher sensitivity to stimulation is reported at higher sinusoidal frequencies, but the phase relationships are highly variable between studies^4,5,7,31^. Such discrepancies may reflect compounding differences in both DC field orientation and fiber response properties across the experimental preparations and animals^5,7^.

The most significant insight from this study is that spike rates in the peripheral vestibular epithelia adapt in response to sustained iDC. The possibility for peripheral adaptation has been identified before for galvanic stimuli^3,6^, but because galvanic stimulation has focused on short pulsed or sinusoidal currents, this effect has not been previously characterized. While it has been shown that central systems adapt in response to repetitive galvanic currents^6,32^, the *ex-vivo* crista preparation suggests that there may also be a peripheral mechanism of adaptation as observed here. Intrinsic spike rate adaptation has been characterized in the vestibular nucleus^23^ with a similar range of time constants and high-pass filter characteristics. This suggests that similar dynamics may be at play in the vestibular periphery. Peripheral adaptation could be due to iDC effects on hair cells^4^, synaptic transmission, and/or afferent nerve fibers directly. Although the somata and central branches of efferent fibers have been cut from the *ex-vivo* crista preparation, peripheral efferent fibers still function and can release neurotransmitter onto hair cells and afferents^33^. iDC stimulation effects via efferent fibers therefore also have to be considered. This highlights the need to understand the effect of electrical stimulation beyond the primary neural target, particularly with stimulation methodologies like DC that may not follow assumptions about neural responses that are based on pulsed stimulation^12^. One resource that may be useful for continued investigation of iDC stimulation effects are the detailed models of field strength and neural effect that have been developed for tDCS experiments in the cortex^16,25,34–36^. An adaptation of these models to account for the unique physiological and morphological properties of the vestibular system would be an invaluable contribution to the understanding of iDC/vestibular afferent interactions.

After iDC stimulation induced adaptation, the vestibular afferents responded with changed sensitivity to subsequent iDC step stimulation at high amplitude. Adaptation to the anodic baseline resulted in greater excitatory responses but reduced inhibitory responses. Adaptation to the cathodic baseline resulted in reduced excitatory responses but no change in inhibitory responses. In the chinchilla, iDC evoked VOR velocities presented a similar trend, albeit also with an increased inhibitory response for cathodic adaptation^9^. The asymmetry in cathodic adaptation response reported here is likely to be an artefact introduced by the low spontaneous rates of the recorded afferent fibers that resulted in a silencing ‘floor’ on afferent activity; of the 10 cells used to analyze adaptation changes, 8 had at least one data point where firing was completely abolished during anodic modulation. Regardless, the *ex-vivo* results suggest that VOR responses observed in the chinchilla can be largely explained by the afferent response to iDC. Combined with other work this suggests that iDC has a fundamentally different effect on the vestibular afferents than pulse-based neuromodulation, where peripheral adaptation was not observed^37^. These data help to explain previous *in-vivo* iDC amplitude-to-behavioral output relationships reported in the chinchilla. This is invaluable for the development of stimulation paradigms in a chronically implanted iDC-based vestibular implant. Moving forward, the *ex-vivo* preparation described here provides an ideal opportunity to explore peripheral mechanisms of DC vestibular modulation via intracellular recordings of the hair cell and afferent fiber membrane potential, pharmacology studies, combined with hair cell or efferent fiber optogenetic activation.

## Materials and Methods

### Animals and preparation

All experimental protocols were carried out according to the guidelines established and approved by the Johns Hopkins Animal Care and Use Committee. Experiments were performed on 32 Wild-Type (WT) C57BL/6J and 5 Mapt-EGFP mice of both sexes, provided by The Jackson Laboratories (USA). Postnatal day 18 to 30 (P18–30) mice were first anesthetized with isoflurane (Forane, Baxter Healthcare Corporation, USA) and then decapitated, and the temporal bone was extracted for removal of inner ear tissue in cold extracellular solution (in mM): 5.8 KCl, 144 NaCl, 1,3 CaCl_2_, 0.9 MgCl_2_, 0.7 NaH_2_PO_4_, 5.6 D-Glucose and 10 Hepes; pH 7.4 (NaOH). The dissected tissue included ampullae, utricle, the superior branch of the vestibular nerve that contains the afferent fibers, and Scarpa’s ganglion including its afferent fiber somata^38^. The membranous labyrinth was then opened above the cristae and utricle and remaining cupulae and otoliths located on top of hair cells were removed. All experiments were performed on the anterior canal of the crista ampullaris. The preparation was secured on a coverslip under a pin, transferred to the recording chamber and viewed with a differential interference contrast (DIC) upright microscope (Examiner D1 microscope, Zeiss, Germany). The microscope was equipped with a 10X and 40X magnification and the images were displayed with an extra 4X magnification on a monitor via a NC70 Newvicon camera (Dage MTI, USA). The recording chamber was constantly perfused with the extracellular solution at the rate of 0.5 ml/min. Prior to entering the recording chamber, the solution was heated at 37 °C using a TC-324B automatic heater control device (Warner Instrument Corporation, USA) with a temperature sensor located in the recording chamber.

### iDC stimulation

iDC stimulation was applied using a ground-isolated precision current source, (Keithley Model 6221 AC and DC Current Source, Tektronix, USA) which was controlled by custom python scripting on an external laptop. The current was delivered using a metal interface (20-gauge steel needle), which was inserted on one side of a Tygon 3350 silicone tube (ABW00002, Saint-Gobain, France) filled with the extracellular solution and gelled in agarose. The needle was positioned far from the tissue (approx. 20 cm) to avoid any contamination caused by the electrolysis at the metal-gel interface. On the opposite side of the tube, a glass-calibrated pipette (Drummond Scientific Company, USA) was inserted (the “iDC stimulator”). The pipettes were pulled with a Model 730 multistep vertical puller (KOPF instrument, USA) in order to obtain a tip diameter of 12.5 µm and filled with the extracellular solution. The return electrode for the iDC stimulator (“iDC reference”) was prepared identically to the iDC stimulator, except the end was connected to a plastic microcatheter pipette (Eppendorf, Germany) filled with extracellular solution and agarose. During the experiments the iDC stimulator was positioned directly across the bath at a 180° angle from the iDC reference at a distance of 0.75 cm and at a 90° angle from the patch pipette, at a distance range of 75–200 µm to the patch pipette (Fig. 1A). The series resistance of the stimulation pathway was 1 MΩ ± 200 kΩ. To focus our experiments on non-capacitive membrane responses during step protocols, a 0.01 µF capacitor was placed in parallel with the circuit to introduce a time constant of 10 ± 2 ms. This capacitor was removed during sinusoidal current modulation.

To investigate the spike rate response to iDC modulation, a step protocol was applied during recording. After recording a 1 min spontaneous rate baseline, 6 sets of 2 second alternating cathodic and anodic steps were delivered at increasing amplitude (Δ 2.5–20 µA). Steps were separated by a 10 second inter-step periods to allow for the cell to recover. This protocol was repeated at the end of the experiment to monitor time-dependent effects, but as no timing effects were observed only the initial protocol was analyzed further.

To analyze the spike rate response to sustained current, iDC was held at ±10 µA for 60 s and followed by the above described cathodic and anodic step protocol, with steps delivered around the new baseline amplitude.

To characterize the frequency response 10 repetitions of sinusoidal ±10 µA current was applied across 7 different frequencies (0.1–8 Hz). During the experimental setup it was confirmed that current across the preparation matched the control signal using a differential probe and isolated oscilloscope TPS 2024B, (Tektronix, Beaverton, OR).

### Loose-patch recordings

Loose-patch recordings were performed using 1 mm borosilicate glass pipettes (WPI, USA), which were pulled with a multistep horizontal puller (Sutter Instruments, USA) in order to obtain a pipette resistance of 4–7 MΩ. The recording pipette was positioned at a 180° angle from the patch reference and at a 90° angle from the iDC stimulator/reference. The pipettes were filled with extracellular solution and recordings were obtained by applying suction to the external wall of the calyx, which increased the seal resistance to 30–80 MΩ. The extracellular signal of vestibular nerve activity was recorded in current clamp mode (I = 0) using pCLAMP 10.2 software with a Multiclamp 700B amplifier (Molecular Devices, USA), sampled at 50 kHz by a Digidata 1322 A (Molecular Devices, USA) and low-pass filtered at 10 kHz.

### Data analysis

The extracellular spikes were detected using Clampfit (Molecular Devices, USA) and MiniAnalysis (Synaptosoft; RRID:SCR_014441). In addition, all the events were double-checked by eye. The data analysis was performed using GraphPad Prism v.6 (USA) and custom Visual Basic scripting in Excel (Microsoft, USA). Average spike rate was defined as the number of spikes in a bin divided by the bin period. Spontaneous rates were determined by taking the average spike rate across a 60 second period without stimulation. The first 50 ms post-stimulus response was blanked during analysis due to the presence of a large stimulation artefact that could interfere with spike analysis. The onset response to iDC was therefore defined as the spike rate during the first 50–550 ms following the stimulus artefact. The action potential waveform maintained the same characteristic shape during the analysis window for both onset and sustained responses. The sustained response to iDC was defined as the average spike rate from 10 to 60 s following the stimulus artefact. Change in spike rate was defined as the average spike rate minus the spontaneous rate. Coefficient of variation (CV) was defined as the standard deviation of the inter-spike intervals divided by the mean interval. Responses were not fit to sinusoidal stimulation, as the evoked spike rate was not strongly sinusoidal and results had a high error. Instead, the excitatory response to sinusoidal modulation was defined as a 180° angle around the middle spike of the response. The other 180° was considered the inhibitory phase. Phase lead or lag was defined as the phase difference between the timing of the middle spike and the peak of the waveform, with positive values corresponding to phase lead and negative values to phase lag.

All data were analyzed via a Shapiro-Wilk test for normality. Non-parametric data were analyzed via a repeated-measures Friedman test or Wilcoxon test. Parametric data were analyzed using a paired t-test or a 2-way repeated measure analysis of variance (2-way RM ANOVA). A Holm-Sidak’s or Dunn’s multiple comparisons test was used for non-parametric or parametric post-hoc analysis, respectively. Correlations were determined using a Pearson’s R test. To determine the rate of adaptation, instantaneous spike rate (1/inter-event interval) over time was fitted with one-phase and two-phase exponential. The simpler fit was determined using an extra sum-of-squares F test. The time constant of spike rate adaptation was defined as the time required for this fit to reach 63.2% of the plateau. Results are expressed as mean ± standard error of the mean (SEM). In all tests, statistical significance was defined as p < 0.05.
